# Detection of Hepatitis B Virus (HBV) Genomes and HBV Drug Resistant Variants by Deep Sequencing Analysis of HBV Genomes in Immune Cell Subsets of HBV Mono-Infected and/or Human Immunodeficiency Virus Type-1 (HIV-1) and HBV Co-Infected Individuals

**DOI:** 10.1371/journal.pone.0137568

**Published:** 2015-09-21

**Authors:** Z. Lee, S. Nishikawa, S. Gao, J. B. Eksteen, M. Czub, M. J. Gill, C. Osiowy, F. van der Meer, G. van Marle, C. S. Coffin

**Affiliations:** 1 Liver Unit, Division of Gastroenterology and Hepatology, Cumming School of Medicine, University of Calgary, Calgary, AB, Canada; 2 Department of Microbiology, Immunology and Infectious Diseases, Cumming School of Medicine, University of Calgary, Calgary, AB, Canada; 3 Department of Comparative Biology and Experimental Medicine, Faculty of Veterinary Medicine, University of Calgary, Calgary, AB, Canada; 4 Bloodborne Pathogens and Hepatitis Laboratory of the National Microbiology Laboratory, Winnipeg, MB, Canada; 5 Ecosystem and Public Health, Faculty of Veterinary Medicine, University of Calgary, Calgary, AB, Canada; University of Cincinnati College of Medicine, UNITED STATES

## Abstract

The hepatitis B virus (HBV) and the human immunodeficiency virus type 1 (HIV-1) can infect cells of the lymphatic system. It is unknown whether HIV-1 co-infection impacts infection of peripheral blood mononuclear cell (PBMC) subsets by the HBV. **Aims** To compare the detection of HBV genomes and HBV sequences in unsorted PBMCs and subsets (i.e., CD4+ T, CD8+ T, CD14+ monocytes, CD19+ B, CD56+ NK cells) in HBV mono-infected vs. HBV/HIV-1 co-infected individuals. **Methods** Total PBMC and subsets isolated from 14 HBV mono-infected (4/14 before and after anti-HBV therapy) and 6 HBV/HIV-1 co-infected individuals (5/6 consistently on dual active anti-HBV/HIV therapy) were tested for HBV genomes, including replication indicative HBV covalently closed circular (ccc)-DNA, by nested PCR/nucleic hybridization and/or quantitative PCR. In CD4+, and/or CD56+ subsets from two HBV monoinfected cases, the HBV polymerase/overlapping surface region was analyzed by next generation sequencing. **Results** All analyzed whole PBMC from HBV monoinfected and HBV/HIV coinfected individuals were HBV genome positive. Similarly, HBV DNA was detected in all target PBMC subsets regardless of antiviral therapy, but was absent from the CD4+ T cell subset from all HBV/HIV-1 positive cases (*P*<0.04). In the CD4+ and CD56+ subset of 2 HBV monoinfected cases on tenofovir therapy, mutations at residues associated with drug resistance and/or immune escape (i.e., G145R) were detected in a minor percentage of the population. **Summary** HBV genomes and drug resistant variants were detectable in PBMC subsets from HBV mono-infected individuals. The HBV replicates in PBMC subsets of HBV/HIV-1 patients except the CD4+ T cell subpopulation.

## Introduction

Hepatitis B virus (HBV) and human immunodeficiency virus type 1 (HIV-1) co-infection is common due to shared modes of transmission with an estimated 4 million co-infected people worldwide [1]. Compared to HBV mono-infected patients, chronic hepatitis B and HIV-1 co-infection increases the risk of end-stage liver disease and the development of cirrhosis and primary liver cancer or hepatocellular carcinoma [2–4].

Although HBV is predominantly a hepatotropic virus, it has been shown to infect lymphoid cells [5]. In the closely related woodchuck animal model of HBV, woodchuck hepatitis virus (WHV) infection can be completely restricted to the lymphatic system and WHV invasion of lymphoid cells is related to the viral load [6,7]. In human studies, HBV genomes are detectable in peripheral blood mononuclear cells (PBMC)s from HBV mono-infected patients despite suppressive anti-HBV nucleos/tide analog (NA) therapy [8], in patients after resolution of acute hepatitis B with HBV surface antigen (HBsAg) clearance [9,10], and in circulating transplacental PBMC from HBV positive mothers possibly leading to *in utero* infection of the neonate [11]. Productive HBV replication is evidenced by the detection of HBV antigens, messenger RNA (mRNA), HBV covalently closed circular DNA (cccDNA) and integrated forms in PBMC and extrahepatic tissues such as, bone marrow cells, spleen, and lymphoblastoid cell lines [12,13]. Additionally, upregulation of HBV replication in PBMC occurs following *ex-vivo* mitogen stimulation and the release of viral particles capable of further infection and replication from these HBV infected PBMC [14]. HBV genomes and viral proteins have been detected within a variety of immune cell subpopulations and in some reports the virus appears to specifically target B cells and monocytes [15–18]. The pathogenic relevance of HBV lymphotropism is unknown, but epidemiological studies suggest an increased risk of lymphatic disorders including chronic lymphocytic leukemia and Non-Hodgkins lymphoma [19–22]. Moreover, unique HBV variants in PBMCs, including immune escape mutants, have been linked to vaccine failure and recurrence of HBV infection after liver transplant [23–27].

In HBV/HIV-1 co-infected patients, HBV genomes, replicative forms, and viral antigens have been detected within total PBMC [28–30]. The HIV-1 primarily replicates within CD4+ T lymphocytes but can also infect myeloid cells, including macrophages and dendritic cells leading to the acquired immunodeficiency syndrome (HIV/AIDS). HBV immune cell co-infection may reflect the HBV immune status, disease phase, as well as the risk of HIV-1 related disease including lymphoproliferative disorders [31]. Few studies have evaluated HBV carriage and genome carriage within PBMC and specific immune cell subsets of HBV monoinfected or in HBV/HIV-1 positive patients on potent NA therapy targeting the HBV polymerase. We hypothesize that co-infection with HIV-1 will affect HBV detection in CD4+/CD8+ T cells, CD14+ monocytes, CD19+ B and/or CD56+ NK cells as compared to HBV mono-infection.

## Results

### Summary of Patient clinical and virological data (Table 1)

In total, 14 treatment naïve HBV mono-infected patients and 6 HBV-HIV co-infected patients, 5/6 on highly active antiretroviral therapy (HAART) were enrolled. All patients were HCV antibody negative. HBV genotyping was available in 3 HBV monoinfected cases and found to have HBV genotype B (ID#1) and C (ID#3), and D (ID#8). At the time of enrolment, 7/14 HBV mono-infected were HBV e antigen (HBeAg) positive (+) / anti-HBe-negative (-) with a median plasma HBV DNA of 5.4 X 10^5^ IU mL-1 (<20–3.6 x 10^7^ IU mL-1 or ∼100–1.8 x 10^6^ virus copies mL-1), median alanine aminotransferase of 47.5 IU L-1 (range 23–236 IU L-1), and 3/14 had moderate to severe liver fibrosis by transient elastography or liver stiffness measurement. Follow-up blood samples were collected from 5/14 HBV mono-infected cases, of which 4/5 had started anti-HBV therapy (e.g. tenofovir or entecavir, median duration 22.6 months, range 16–32) with suppressed plasma HBV DNA as determined by a kinetic PCR assay (COBAS TaqMan HBV, Roche Molecular Systems). In addition, 6 HBV-HIV co-infected patients were enrolled. In the HBV/HIV-1 co-infected cohort, 3/6 were HBeAg(+)/anti-HBe(-), 5/6 were consistently treated with highly active antiretroviral therapy (HAART), with median HBV DNA 313 IU mL-1 (<55–690 IU mL-1 or ~ 300–3.5 x 10^3^ virus copies mL-1), median ALT 43 (range 15–54 IU L-1), median CD4+ T cell count 240 cells/mm^3^ (114–800 cells mm^3^–1) and median HIV-1 RNA <40 copies mL-1 (<40–10^4^ copies mL-1). One HBV/HIV-1 co-infected patient was intermittently compliant with antiviral therapy (HBV-HIV ID#3) and had low-level plasma HBV viremia (<100 copies mL-1). In the co-infected cases the HBV genotype was not done or could not be determined by line probe assay, especially if the patient was on suppressive antiviral therapy with suppressed HBV DNA In plasma. Overall, both the HBV mono-infected and HBV/HIV-1 co-infected cohorts had comparable clinical parameters except for significantly lower plasma HBV DNA levels in the HBV/HIV-1 co-infected cohort on HAART as compared to the HBV mono-infected treatment naïve cohort (*P* < 0.01) (**Table 1**). The difference in HBV DNA detection in plasma in the treatment naïve HBV monoinfected versus HBV/HIV coinfected is likely due to the effect of HAART with dual anti-HBV/HIV activity (i.e., Tenofovir, Lamivudine or Emtricitabine).

**Table 1:** Summary of clinical information from 6 HBV/HIV-1 coinfected and 14 HBV mono-infected patients.

Variable	HBV Mono-infected	HBV/HIV-1 Coinfected
Sex N = M/F	14 = 13M/1F ^a^	6 = 5M/1F
Median Age, years (range)	46.2 (26–62)	45 (22–60)
HBeAg Positive, N	7/14	3/6
Median HBV DNA, IU/mL (range) ^b^	Baseline: 5.4 x 10^5^ (<20–3.6 x 10^7^); Follow up: 3.6 x 10^2^ (<10–1.7 x 10^3^)	313 (<55–690)
Median alanine aminotransferase (IU/L) (range)	47.5 (23–236)	43 (15–54)
Median CD4+ T cell (cells/mm^3^) (range)	N/A ^c^	240 (114–800)
Median HIV RNA, copies/mL (range) ^d^	N/A	<40 (<40–10^4^)
Liver Fibrosis Stage by Transient Elastography, N	Stage 0–1 (N = 8), Stage 2 (N = 4), Stage 3–4 (N = 2)	Unknown
Antiviral Treatment	14/14 baseline Rx naïve, 1/5 follow up Rx naïve, 4/5 follow up on anti-HBV Rx	5/6 on HAART with anti-HBV activity^e^

^a^ 5/14 follow-up samples (4/5 on therapy).

^b^ HBV DNA tested using kinetic PCR (sensitivity <20 or <55 IU/mL, 100–300 copies/mL; TaqMan, Roche).

^c^ normal CD4+ T cell count is 500 cells/mm^3^ to 1,000 cells/mm^3^.

^d^ HIV RNA tested using real time PCR assay with sensitivity <40 or 75–10^10^ virus copies/mL (Abbott m2000).

^e^ HAART—highly active antiretroviral therapy.

### Detection of HBV genomes in whole PBMC and cell subsets from HBV mono-infected patients (Table 2)

**Fig 1:**
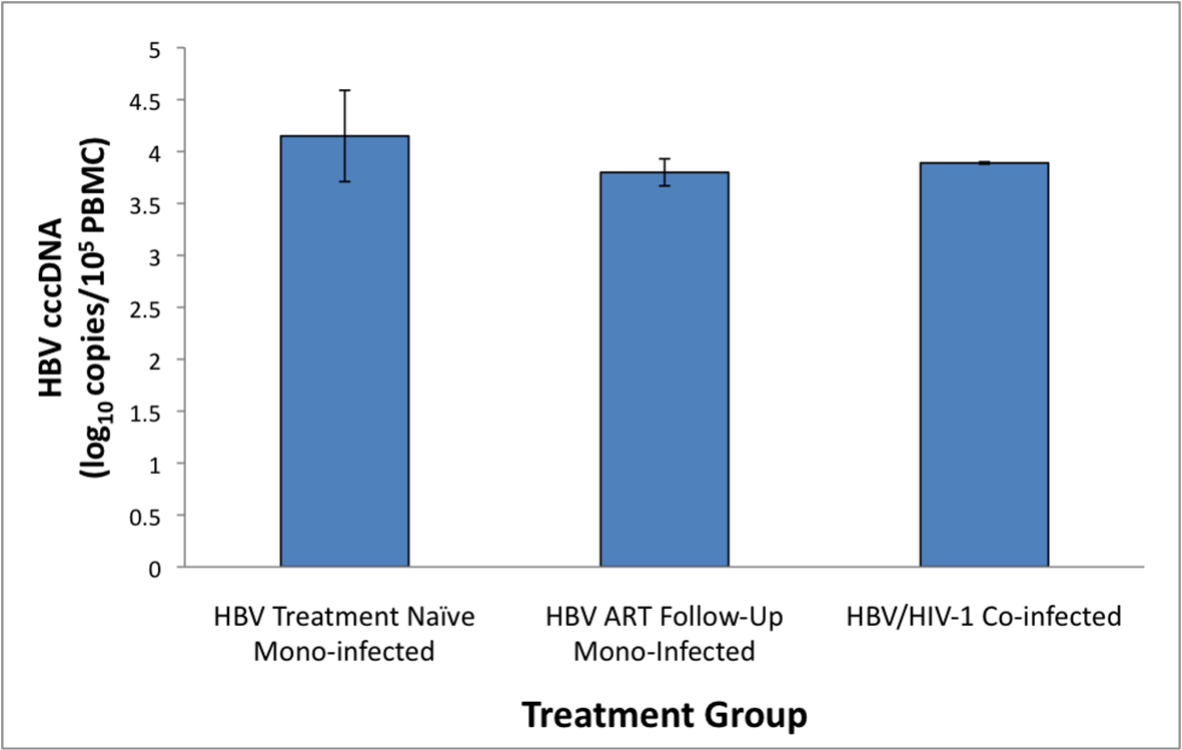
Comparison of median HBV covalently closed circular (ccc) DNA copies in peripheral blood mononuclear cells in HBV mono-infected (before and after antiviral treatment) and HBV/HIV-1 co-infected patients. The HBV covalently closed circular (ccc)—DNA copies/peripheral blood mononuclear cell (PBMC) were determined by quantitative PCR using a TaqMan probe and normalized to a housekeeping gene (i.e., β-globin, β-glo). The HBV cccDNA copies/PBMC did not significantly differ between groups, i.e, HBV treatment naïve mono-infected group (n = 11): median 4.2, range 3.4–4.7 log10 copies/10^5^ PBMC; HBV mono-infected on antiviral therapy (n = 4): median 3.8, range 3.6–3.9 log10 copies/10^5^ PBMC; and HBV/HIV-1 co-infected (n = 2) mean 3.8 copies/10^5^ PBMC.

HBV DNA and/or replication-indicative HBV mRNA and cccDNA were detected in unsorted PBMC isolated from 13/13 treatment naïve HBV mono-infected patients at baseline and in all HBV monoinfected patients that subsequently started on anti-HBV NA (ID#1, 3, 8, 13) **(Table 2, S2 Fig)**. HBV genome analysis in specific immune cell subsets from either treatment naïve or antiviral treated HBV mono-infected patients showed that HBV DNA was detectable in all subset types regardless of whether HBV replication in their plasma was suppressed with highly potent anti-HBV nucleos/tide analog therapy. Thus, there was no significant difference in the frequency of HBV DNA detection between cell subsets isolated from treatment naïve compared to antiviral treated HBV mono-infected patients. The median HBV-cccDNA log copy number in whole PBMC in treatment naïve HBV mono-infected patients was 4.2 (range 3.45–4.72 log10 copies/10^5^ PBMC), and did not significantly differ from the median HBV cccDNA copy number in HBV patients after starting on anti-HBV treatment (median 3.8, range 3.6–3.9 log10 copies/10^5^ PBMC) (*P* = 0.09) (**Fig 1**).

**Table 2:** Summary of HBV genome detection in whole peripheral blood mononuclear cells and subsets isolated from 14 HBV mono-infected patients.

HBV Mono-infected ID#	PBMC Subset (HBV DNA) ^a^					Whole PBMC		
	CD4	CD8	CD14	CD19	CD56	DNA	mRNA	ccc DNA
1A	+	-	-	+	+	+	+	+
1B	-	N/A	+	N/A	N/A	+	+	+
2	+	-	-	-	-	+	+	+
3A	+	-	-	+	+	+	+	+
3B	+	+	+	N/A	+	+	+	+
4	-	-	+	+	+	+	+	+
5	N/A	-	-	+	-	+	+	+
6	+	+	-	+	N/A	+	+	+
7	+	-	+	-	-	+	+	N/A
8A	+	-	-	+	+	+	+	+
8B	+	-	+	+	+	+	+	+
9	-	+	-	-	+	N/A	+	N/A
10	+	+	+	N/A	+	N/A	N/A	N/A
11	N/A	N/A	-	-	-	+	+	+
12A	+	+	-	N/A	-	+	+	+
12B ^b^	+	N/A	+	N/A	-	+	+	+
13A	-	+	+	-	+	+	+	+
13B	+	+	-	N/A	N/A	+	+	+
14	-	-	+	-	-	+	+	+
Summary: HBV DNA+ Treatment Naïve, N	8/12	5/13	5/14	6/13	7/13	12/12	13/13	11/11
Summary: HBV DNA+, antiviral therapy ^c^, N	3/3	2/3	3/4	1/1	2/2	4/4	4/4	4/4

^a^ HBV DNA tested using HBV specific Core, Surface, and Polymerase primers. All subsets from HBV mono-infected cases tested HBV cccDNA negative.

^b^ ID# 12B –follow up sample collected but patient treatment naïve.

^c^ Follow up patient samples collected from # 1B, 3B, 8B, 13B after initiation of antivirals, all tested HBV DNA negative in serum according to a real-time PCR.

N/A–samples excluded from PCR analysis if <80% cell purity by Flow Cytometry analysis or insufficient template. In some cases, all the whole PBMC were seperated into cell fractions and there were no unsorted cells left-over available to test for HBV cccDNA.

### Detection of HBV genomes in whole PBMC and individual cell subsets from HBV/HIV coinfected patients (Table 3)

In 6/6 HBV/HIV-1 coinfected cases tested (5/6 on HAART and 1/6 intermittent compliance with therapy) HBV DNA was found in at least one cell subset with the exception of the CD4+ T cell subpopulation (*P*<0.04) **(Table 3)**. HBV cccDNA was also detected in CD8+ T and CD56+ NK cell subsets from 3/6 HBV/HIV-1 co-infected patients. As confirmation, HBV mRNA and HBV cccDNA was readily detectable in whole PBMC available from 2 HBV/HIV-1 coinfected patients on HAART. HBV cccDNA quantification in the whole PBMC showed the median log copy number was 3.8 (3.8–3.9 log10 copies/10^5^ PBMC), which was compatible to the cccDNA load found in whole PBMC of HBV mono-infected patients on anti-HBV NA therapy (**Fig 1**).

**Table 3:** Summary of HBV genome detection in whole peripheral blood mononuclear cells and subsets isolated from 6 HBV/HIV-1 coinfected patients.

HBV/HIV-1 Co-infected ID#	PBMC Subset HBV DNA and (*cccDNA*) ^a^					Whole PBMC		
	CD4+	CD8+	CD14+	CD19+	CD56+	DNA	mRNA	cccDNA
1	-	+ **(-)**	-	-	N/A	+	+	**+**
2	-	-	+	N/A	-	N/A	N/A	N/A
3^b^	-	+(**+)**	+	+	+(**+)**	+	+	**+**
4	-	-	-	-	+(**+)**	N/A	N/A	N/A
5	-	+(**+)**	-	-	+(**+)**	N/A	N/A	N/A
6	N/A	-	+ **(-)**	+ **(-)**	+	N/A	N/A	N/A
Summary: HBV DNA and/or ccc-DNA positive on HAART ^b^	0/5 (*P*<0.04)	3/6	3/6	2/5	4/5	2/2	2/2	2/2

^a^ HBV DNA tested using HBV specific Core, Surface, and Polymerase primers.

^b^ HBV/HIV positive Case ID#3 intermittent compliance with antiretroviral therapy.

**bold** font in brackets indicates cccDNA test results. N/A–samples excluded from summary analysis if <80% cell purity by FACS. In most cases, all of the PBMC collected was separated into cell fractions and thus analysis of unsorted (whole PBMC) was not possible.

### Detection of HBV Drug Resistant and Immune Escape Variants in HBV Monoinfected Immune Cell Subsets by Next Generation Sequencing Analysis (Fig 2, S1 Table)

**Fig 2:**
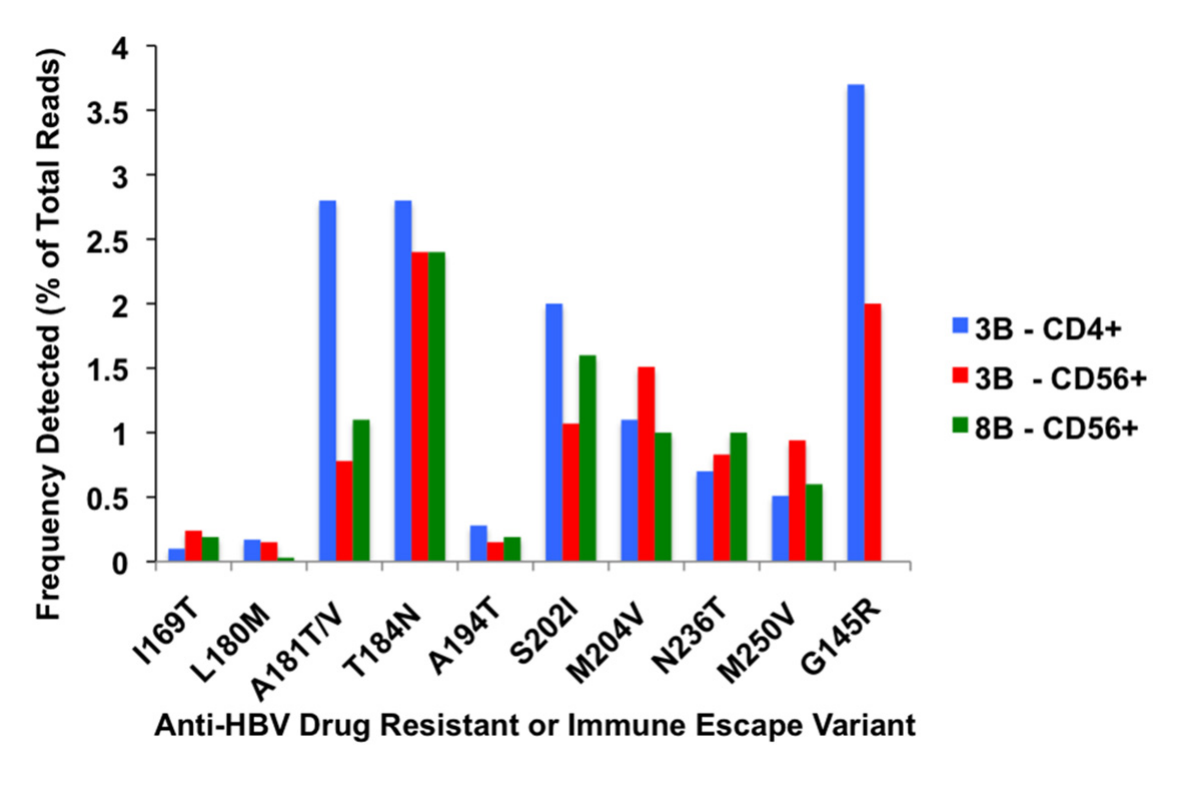
Frequency (% of total reads) of HBV drug-resistant and immune escape (i.e., G145R) mutations detected in CD4+ and/or CD56+ immune cell subset by deep sequencing analysis in 2 HBV monoinfected cases (i.e., #3B and #8B) on tenofovir anti-HBV therapy.

Deep sequencing analysis of HBV P/overlapping S gene sequences in 2 HBV monoinfected patients after starting potent anti-HBV therapy (Case #3B and #8B) in CD4+ and/or CD56+ compartment showed the presence of drug resistant mutations in a minor percentage of the population (range 0.03–3.8%). Both cases were on tenofovir therapy with undetectable HBV DNA in plasma according to clinical PCR assay. In comparision, Sanger sequence analysis (~7 clones per sample) of each case #3B-CD56+ and #8B-CD56+) showed only wild type sequences at these sites. Error rate estimation was determined by alignment and comparison of nucleotide substitution prevalence in the NGS sequences from a plasmid clone (PCR amplified with NGS adaptor tags) to Sanger-sequenced plasmid clones. A low prevalence of drug resistance mutations was observed in the quasispecies of CD56+ samples from Case #3B and #8B, however this was no fold—difference from the estimated error rate at each mutation site (S1 Table). Case #3B also carried the classic HBV vaccine escape variant (i.e, G145R) in the CD4+ and CD56+ compartment at ~2–4% of the total population. In the CD56+ sample of Case #3B, the G145R variant was found in greater frequency in the plasmid control NGS data compared to the percentage of mutation in the matching sample. Thus to determine the true prevalence of immune escape variants within immune cell subsets requires confirmation in future studies with more clinical samples (**Fig 2, S1 Table**) (GenBank Accession # pending).

## Discussion

The aim of this study was to compare the presence of HBV genomes within whole (unsorted PBMC) and immune cell subsets from HBV mono-infected and/or HBV/HIV-1 coinfected individuals. In the current study we found HBV DNA, HBV cccDNA and mRNA in both whole (unsorted) PBMC isolated from HBV mono-infected (either treatment naïve and after starting anti-HBV therapy) and in HBV/HIV-1 co-infected patients on dual-active anti-HBV/HIV therapy. In the current cohorts, HBV genomes were detected in all PBMC subset types, with the exception of the CD4+ T cell subpopulation in HBV/HIV-1 co-infected cases. Although Sanger sequencing analysis revealed only wild-type HBV at residues associated with drug resistance or immune escape, deep sequencing analysis of viral sequences in immune cell subsets (CD4+ and CD56+) from 2 HBV monoinfected cases (#3B and #8B) on tenofovir antiviral therapy revealed the presence of drug resistant variants in a minor percentage of the population. In Case #3B, the classic vaccine escape mutant (i.e., G145R) was also detected in both the CD4+ T cell and the CD56+ T cell subset, albeit in greater frequency in the estimated error rate based on the NGS-sequenced plasmid clone of the CD56+ samples (S1 Table), thus the data requires confirmation in further studies.

Earlier studies show conflicting data with regard to infection of PBMC subsets by HBV. In HBV mono-infected patients, HBV genomes were detected in all PBMC subsets, with no significant difference in frequency [16]. Pasquinelli et al. detected HBV genomes but no replicative forms in CD4+ and CD8+ T cells and B lymphocytes using a less sensitivie ^32^P-labelled PCR probe hybridization assay (sensitivity ~4 x 10^8^ virus copies μg-1 HBV DNA) [32]. Yoffe et al. (using a similar less-sensitive hybridization assay) also detected HBV DNA in monocytes and B cells, but HBV genomes were not found in the T and NK cell subset [15]. The discrepancy between previous reports and the current study may be due to differences in PBMC subset isolation and enrichment techniques, the enhanced sensitivity of our nested PCR/NAH and quantitative PCR assay and the use of multiple HBV primers to detect HBV genomes. All HBV mono-infected cell subsets did test negative for HBV cccDNA, despite testing positive for HBV genomes using HBV C, S and P specific primers, and as well as testing positive for HBV cccDNA in whole unsorted PBMC. This may be related to the limited template remaining from the individual cell subsets to test for HBV cccDNA (after testing for HBV C, S and P specific gene fragments), as well as the overall low predominance of HBV cccDNA overall, affecting detection after cell sorting.

In the current study, HBV genomes were detectable in PBMC, despite suppression of HBV DNA in plasma (following initiation of anti-HBV NA in HBV monoinfected cases or in HBV/HIV co-infected patients already on HARRT). This data is in agreement with our prior studies showing persistence of the HBV genome, minor drug resistant, and possible immune escape variants within PBMC in treatment naïve HBV infected individuals, patients on potent anti-HBV therapy or in liver transplant recipients on HBV prophylaxis [8,25,33]. Other studies have also described detection of the classic vaccine escape mutant (i.e., G145R) in the PBMC compartment that was linked to vertical and intrafamilial horizontal occult (i.e., low-level) HBV transmission [23]. Additionally, our studies in HIV-1 positive patients with occult hepatitis B infection on HAART clearly demonstrated the presence of HBV DNA and HBV ccc-DNA in PBMC even despite absence of HBV DNA in the plasma compartment [34]. In the current study, replicative-indicative HBV cccDNA and/or mRNA was detected in unsorted PBMC of HBV mono-infected patients as well as unsorted PBMC and CD8+ T and CD56+ NK cells of HBV/HIV-1 coinfected patients. The detection of HBV mRNA and cccDNA within unsorted PBMCs and subsets suggests active HBV viral replication in the lymphoid cell compartment [35]. Moreover, our recent work suggest that the unique biological conditions within the PBMC, may allow HBV to persist and evolve differently within the PBMC compared to the plasma or liver site [33]. HIV-1 co-infection may in turn change the biology of PBMC subsets and alter their susceptibility to HBV infection.

The mechanisms by which HIV-1 infection can potentially impact HBV infection in specific PBMC subsets remain unclear. A study by Stacey et al. [36], documented that plasma cytokine profiles of HIV-1 positive individuals as compared to HBV mono-infected individuals showed sustained increases of IFN-α and monocyte chemotactic protein 1, lower increases of proinflammatory factors such as interferon gamma (IFN-γ) and late increases in immunoregulatory cytokines like interleukin 10 (IL-10). Increased T-cell turnover and proinflammatory cytokines observed during HIV-1 infection might alter the cytokine milieu affecting the susceptibility of PBMCs to HBV infection. Alternatively, HIV-1 and HBV may directly compete for host cellular machinery. For example, the entry binding proteins for HBV (S proteins) and the HIV-1 envelope (Env, gp160) are membrane-associated proteins, and could interfere with each other in the assembly process in HBV/HIV coinfected cells [37]. However, the possibility that one virus would directly exclude infection by the other, would be unexpected given that the number of HIV infected CD4+ T cells in the periphery is considered to be relatively low (i.e., ~1.4% of the total subpopulation) [38].

The detection of false positive HBV genome signals is unlikely, as all experiments included strict contamination controls and lab procedural precautions, and HBV DNA was not detected in every PBMC subsets from individual patient samples. The inability to detect HBV genomes in CD4+ T cells from HBV/HIV-1 coinfected samples is also not explained by the potentially lower levels of CD4+ T cells in HIV-1 coinfected patients enrolled in this study. The median CD4+ T cell count of the HBV/HIV-1 cohort was 240 cells mm^3^-1 (113–800 cells mm^3^-1) and although CD4+ T cell counts were not determined in HBV mono-infected cohorts, the expected normal range in HIV-1 negative individuals is ~500–1,000 cells mm^3^-1 [39,40]. In all assays the target subset fractions were enriched and input HBV DNA template for PCR was standardized. We acknowledge that future studies with additional clinical samples are planned to confirm these findings.

In summary, the HBV can be detected in whole PBMC and in most immune cell subsets of HBV mono-infected and HBV/HIV co-infected patients even despite potent anti-HBV nucleoside analog therapy, including the presence of drug resistant and potentially immune escape variants in a minor percentage of the population. However, the HBV was excluded from CD4+ T cells of all HBV/HIV-1 co-infected patients tested. The study provides additional evidence supporting the role for PBMCs to act as a viral reservoir for the HBV, and suggests that concomitant HIV-1 infection affects HBV lymphotropism. HBV immune system infection may have clinical relevance regarding the natural history of HBV-related liver and extrahepatic disease in patients co-infected with HIV.

## Materials and Methods

### Patients, Clinical and Laboratory Tests

The local institutional review board (i.e., the University of Calgary conjoint health research ethics board, CHREB) specifically approved the study (Ethics ID #16636 and #3220). All subjects provided informed written consent according to the World Medical Association Declaration of Helsinki. In total, 14 HBV mono-infected (13 M/1F, median age 46.2 y, range 26–62), and 6 HBV/HIV-1 co-infected (5M/1F, median age 45 y, range 22–60) patients were enrolled in our study (**Table 1**). All patients tested hepatitis C virus (HCV) antibody negative. HBV genotyping was done in 3 HBV monoinfected cases by INNO-LiPA line probe assay (Innogenetics, N.V., Ghent, Belgium). Chronic hepatitis B or HIV-1 co-infection was diagnosed according to standard clinical, virological and serological criteria. The clinical and demographic data collected included age, sex, co-morbid liver disease and antiviral therapy. Laboratory information included CD4+ T cell count, HIV-1 and HBV plasma viral load, liver enzymes, and platelet count. Transient elastography (FibroScan®, Echosens, France) was used to assess stages of liver fibrosis. HBV serologic markers included HBsAg, HBV e antigen (HBeAg), hepatitis B surface antibody (anti-HBs), and HBe antibody (anti-HBe), were evaluated by commercial chemiluminescent microparticle immunoassays (ARCHITECT Anti-HBsAg Qualitative, anti-HBs; Abbott Diagnostics, Mississauga, ON, Canada). The HBV DNA levels in serum were tested by PCR (COBAS TaqMan HBV test, detection limit <20–<55 IU mL-1 or ∼100–300 copies mL-1, Roche Molecular Systems, Inc., Branchburg, NJ, USA). The HIV-1 RNA was tested using the Abbott Real Time HIV-1 assay m2000 (<40–10^7^ virus copies mL-1, Abbott, Mississauga, ON, Canada).

### Whole PBMC Isolation, Cell Subset Purification, and Fluorescence-Activated Cell Sorting Analysis (FACS)

Whole blood (~60–80 ml) was obtained from patients (N = 20) and healthy volunteers (N = 7). Plasma was collected and PBMC were isolated on a Ficoll gradient. Approximately 2.0 x 10^7^ of whole PBMC were cryopreserved and the remaining cells divided into equal aliquots for the positive selection of CD4+ T cells, CD8+ T cells, CD14+ monocytes, CD19+ B cells and CD56+ NK cells with magnetic bead monoclonal human antibodies as per the manufacturer’s instructions (MACS Miltenyi Biotec®, Auburn, CA, USA). The purity of the cell subsets (~ 10^6^ cells from each fraction) were verified using dual color immunofluorescence flow cytometry (BD Biosciences LSR II, San Jose, California) and analyzed with Kaluza software (Beckman Coulter, Mississauga, Ontario) (**S2 Fig**). During all procedures, precautions were undertaken to avoid cross-contamination and to standardize cell-sorting experiments. Samples with <80% purity as determined by FACS analysis, were excluded in the final data analysis. The final PBMC wash after isolation and the left—over flow-through following magnetic bead cell separation was saved for PCR analysis (data not shown).

### Nucleic acid isolation and detection of HBV genomes

Total PBMC and cell subsets were treated with DNase/trypsin to remove any adhering extracellular viral particles [9]. Total DNA and RNA were isolated using commercial nucleic acid extraction kits (Illustra TriplePrep kit, GE Healthcare, Chalfont St Giles, Buckinghamshire), and/or by standard phenol-chloroform/ethanol precipitation method. Total RNA was isolated from whole PBMC (13/14 HBV mono-infected and 3/5 HBV/HIV-1 co-infected) using TRIzol® reagent (Roche technologies, CA, USA). The isolated total PBMC and cell subsets were analyzed for HBV genomes using HBV specific core (C), surface (S), and polymerase (P) primers [8,25,41] via nested PCR/digoxigenin-antidigoxigenin detection (DIG) labelled probe according to the manufacturer’s instructions (Roche Diagnostics, Manheim, Germany). PBMC subsets which tested positive for HBV DNA with at least one HBV gene specific primer were analyzed for HBV cccDNA (lower detection limit <10^2^ virus copies mL-1), as described [25]. For detection of HBV mRNA, reverse transcription (RT) of total RNA to complementary (c)DNA was done followed by nested PCR/NAH as described [9]. In addition, HBV cccDNA was tested in whole PBMC by sensitive quantitative real-time assay (lower detection limit ~92 cccDNA copies mL-1). In brief, a 10-fold serial plasmid dilution with either a HBV cccDNA insert or human β-globin (beta-glo) internal control was used to generate duplicate standard curves. All reactions were set up in triplicate using PerfeCTa® FastMix® II, ROX™ (Quanta Biosciences, Gaithersburg, MD, USA) with specific specific cccDNA primers and a TaqMan labelled probe (5’- GTGCCTTCTCATCTGCCGG-3‘; 5’-GGAAAGAAGTCAGAAGGCAA-3’ and probe AGGCTGTAGGCATAAATTGGT) in parallel with homo β-globin primers and probe (5’-CTGGGCAACGTGCTGGTCTG-3’; 5’-GGCAGAATCCAGATGCTCAAG-3’; probe TGCTGGCCCATCACTTTGGCAA). Rigorous precautions were taken against contamination and all reactions included negative controls consisting of total PBMC and subsets from healthy HIV-1 and HBV negative volunteers, mock water extractions, and reagents. All PCR reaction mixes and samples were set up in different rooms and in laminar flow cabinets to minimize cross contamination with amplicons. The reverse transcription step for RNA detection was carried out in parallel with and without reverse transcriptase enzyme to ensure specificity for cDNA detection and not viral DNA carry-over. The positive control was a HBV genotype A *EcoR1* digested HBV genotype A DNA plasmid (kindly provided by Dr. T.I. Michalak).

### Next Generation and Sanger Sequencing of HBV Genomes Detected in Different HBV Immune Cell Subsets

To analyze HBV sequences carried within a specific immune cell subset for presence of drug resistant and/or immune escape variants, the available remaining PCR amplified HBV genomes from Cases #3B and #8B (after starting tenofovir therapy) were analyzed by Sanger sequencing and next generation sequencing technology. In the other cases, which tested positive, the original PCR template was not available, or re-amplification using NGS adaptor-tag primers was unsuccessful. NGS analysis of available HBV P gene amplicons from Cases #3B CD4+ T cells, #3B-CD56+ T cells and #8B CD56+ T cells (i.e, “clinical samples”),were performed by re-amplification in a nested PCR reaction using high fidelity polymerase (Phusion®, New England Biolabs) and HBV specific Illumina index compatible primers with adaptor tags (i.e., HBV Polymerase forward (nt 141–159) with overhang 5’-TCGTCGGCAGCGTCAGATGTGTATAAGAGACAG CAG GAT TCC TAG GAC CCC TGC-3’ and HBV Polymerase reverse (nt 878–899) with overhang GTCTCGTGGGCTCGGAGATGTGTATAAGAGACAG CCA TGA ART TAA GGG AAT AGC CCC-3’, amplicon size 710 bp), followed by sequencing using the the Illumina-MiSeq platform (Illumina, Inc. San Diego, CA). To estimate the putative error rate of experimentation and the Illumina system, the above amplicons were cloned (~ 7 clones per sample) using the Topo-TA cloning system and all clones underwent Sanger sequencing. In addition, an HBV plasmid clone for each sample was amplified with NGS overhang/adaptor tags for sequencing using the Illumina-MiSeq platform and compared to data from the Sanger sequenced clones by alignment to determine the Illumina-associated error-rate. The average sequencing depth for each plasmid or clinical sample by NGS was 2150x. The assembled HBV Polymerase / overlapping Surface sequences were analyzed with Geneious® software (R8.1.5. Biomatters, Auckland, New Zealand). The estimated error rate was taken into account by determining the fold-difference in nucleotide substitution between the plasmid and clinical sample NGS sequence data (see S1 Table).

### Data Analysis

A nonparametric Mood’s median test was used for the comparison of HBV DNA from whole PBMC using Minitab® 17 software. A multivariate analysis of frequency distribution using the Fisher exact test was used for the comparison of HBV genome detection within PBMC subsets from HBV treatment naïve, HBV ART follow-up and HBV/HIV-1 HAART treated patients with SAS® 9.3 software. The statistical significance between the HBV cccDNA detection within HBV/HIV co-infected versus HBV mono-infected (treatment naïve and on treatment) groups were analyzed using a one-way ANOVA and paired t-test respectively. Pearsons *X* ^2^ test was done to compare the % mutation detection in each PBMC subsets analyzed by NGS compared to Sanger (clonal sequencing). Two-tailed P values of < 0.05 were considered statistically significant for all tests.

## Supporting Information

S1 Fig Detection of HBV DNA in PBMC subsets isolated from a treatment naïve HBV mono-infected case. Representative results illustrating detection of HBV genomes by nested PCR/nucleic acid hybridization to a digoxigenin (DIG) labelled PCR probe labelled with surface (S), core (C) and polymerase (P) primers in immune cell subsets isolated from a treatment naïve HBV mono-infected patient (ID# 8A). HBV DNA was detected in CD4+ T cells and CD19+ B cells using HBV specific C primers and CD56+ NK cells using HBV specific P primers. The size of the expected amplicon is indicated to the right of the panel. Water from second round of amplification (NW) and first round of amplification (DW) and a mock nucleic acid extraction served as negative controls. An HBV genotype A plasmid served as the positive control.
(TIFF)

S2 Fig A representative fluorescence activated cell (FACS) sorting density plots and histogram analysis of immune cell subsets isolated from a HBV mono-infected patient. Fluorescence activated cell sorting purity analysis of peripheral blood mononuclear cell subsets enriched via positive magnetic bead cell sorting (Miltenyi®) isolated from a hepatitis B virus (HBV) mono-infected treatment naïve patient (Patient ID# 9). PBMC subsets determined to have >80% purity when compared to a background sample of unlabelled cells were considered suitable for further HBV genome detection assays. In this representative patient sample, the purity of each isolated subset was determined to be 96.8% for CD4+ T cells, 94.8% for CD8+ T cells, 82.8% for CD14+ monocytes, 89.0% for CD19+ B cells and 83.2% for CD56+ natural killer cells.
(TIFF)

S1 Table Summary of HBV Polymerase/Overlapping S Gene Deep Sequencing Analysis in Immune Cell Subsets Isolated from 2 HBV monoinfected patients on suppressive TDF antiviral Therapy. (DOCX)

## Non standard Abbreviation List

WHV: woodchuck hepatitis virus
PBMC: peripheral blood mononuclear cells
NA: nucleos/tide analog
HBV mRNA: hepatitis B virus messenger RNA
HBV ccc-DNA: hepatitis B virus covalently closed circular DNA
HAART: highly active antiretroviral therapy
HBeAg: HBV e antigen
anti-HBe: HBV e antibody
