# Supplementary figures and images for: Detection of Hepatitis B Virus (HBV) Genomes and HBV Drug Resistant Variants by Deep Sequencing Analysis of HBV Genomes in Immune Cell Subsets of HBV Mono-Infected and/or Human Immunodeficiency Virus Type-1 (HIV-1) and HBV Co-Infected Individuals
Source: PLoS One. 2015 Sep 21;10(9):e0137568. doi: 10.1371/journal.pone.0137568 (PMC4577215; doi:10.1371/journal.pone.0137568)

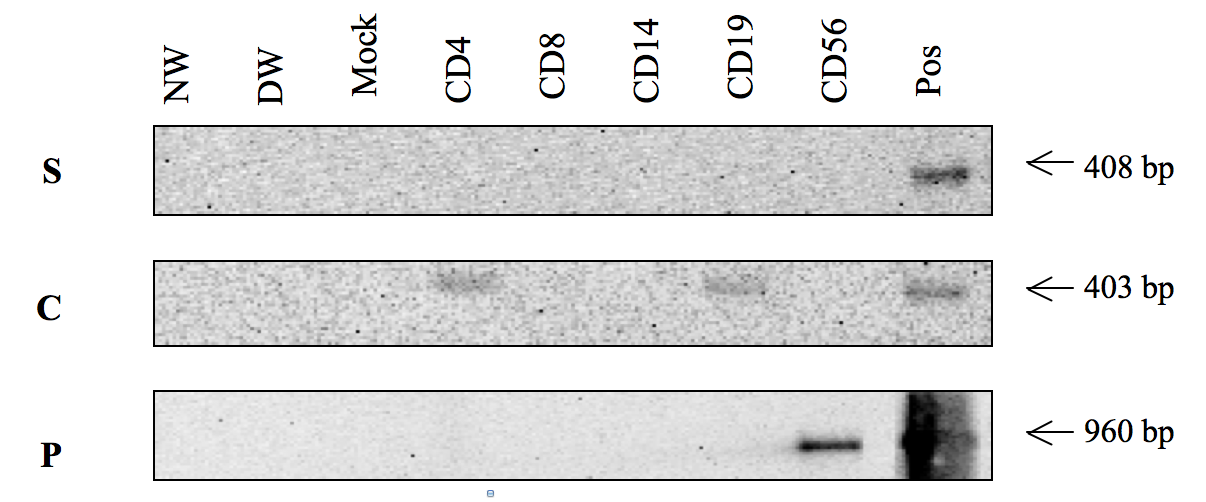

Supplement: S1 Fig — Representative results illustrating detection of HBV genomes by nested PCR/nucleic acid hybridization to a digoxigenin (DIG) labelled PCR probe labelled with surface (S), core (C) and polymerase (P) primers in immune cell subsets isolated from a treatment naïve HBV mono-infected patient (ID# 8A). HBV DNA was detected in CD4+ T cells and CD19+ B cells using HBV specific C primers and CD56+ NK cells using HBV specific P primers. The size of the expected amplicon is indicated to the right of the panel. Water from second round of amplification (NW) and first round of amplification (DW) and a mock nucleic acid extraction served as negative controls. An HBV genotype A plasmid served as the positive control. (TIFF) [file pone.0137568.s001.tiff]

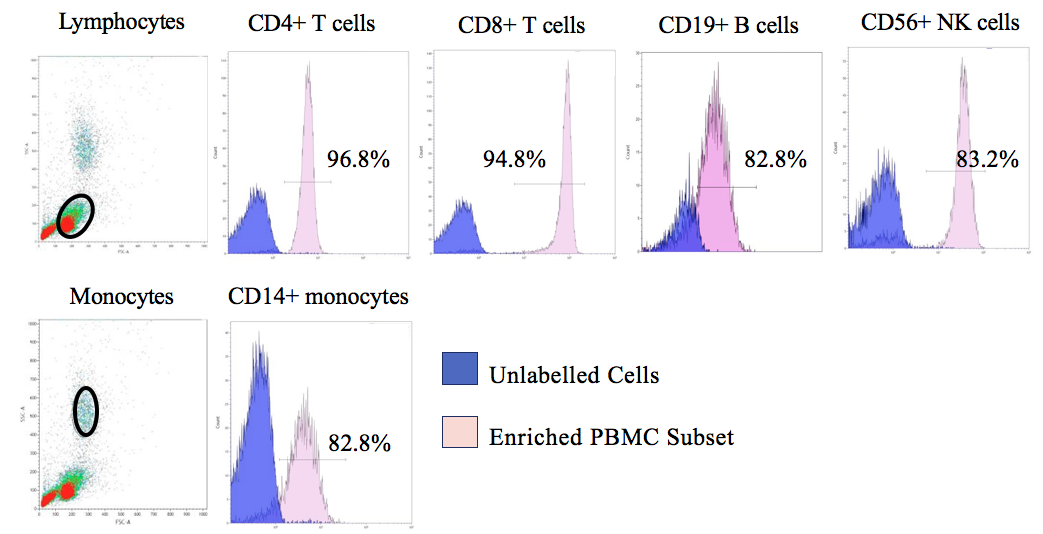

Supplement: S2 Fig — Fluorescence activated cell sorting purity analysis of peripheral blood mononuclear cell subsets enriched via positive magnetic bead cell sorting (Miltenyi®) isolated from a hepatitis B virus (HBV) mono-infected treatment naïve patient (Patient ID# 9). PBMC subsets determined to have >80% purity when compared to a background sample of unlabelled cells were considered suitable for further HBV genome detection assays. In this representative patient sample, the purity of each isolated subset was determined to be 96.8% for CD4+ T cells, 94.8% for CD8+ T cells, 82.8% for CD14+ monocytes, 89.0% for CD19+ B cells and 83.2% for CD56+ natural killer cells. (TIFF) [file pone.0137568.s002.tiff]
