# Supplementary material for: Detection of Hepatitis B Virus (HBV) Genomes and HBV Drug Resistant Variants by Deep Sequencing Analysis of HBV Genomes in Immune Cell Subsets of HBV Mono-Infected and/or Human Immunodeficiency Virus Type-1 (HIV-1) and HBV Co-Infected Individuals
Source: PLoS One. 2015 Sep 21;10(9):e0137568. doi: 10.1371/journal.pone.0137568 (PMC4577215; doi:10.1371/journal.pone.0137568)
Supplement: S1 Table — (DOCX) [file pone.0137568.s003.docx]

**Supplementary Table 1**: Summary of HBV Polymerase/Overlapping S Gene Deep Sequencing Analysis in Immune Cell Subsets Isolated from 2 HBV monoinfected patients on suppressive TDF antiviral Therapy

| Clinical Info /  Patient ID# | Immune Cell Subset | Mutation Polymerase / Drug Resistance and Surface / Immune Escape | % (Number of reads)  vs. Plasmid Control | Fold Difference Compared to Plasmid Control |
| --- | --- | --- | --- | --- |
| 62 Y Asian/M on Tenofovir, Genotype C  #3B | CD4+ | I169T  L180M  T184N  A194T  S202N  M204V/I  N236T  M250I/V  G145R | 0.10 (2/1809)  0.17 (3/1809)  2.80 (52/1809)  0.28 (5/1809)  2.00 (37/1809)  1.10 (21/1809)  0.70 (14/1809)  0.51 (9/1809)  3.70 (237/6480) | N/A (no Plasmid Control) |
|  | CD56+ | I169T  L180M  A181T/V T184N  A194T  S202I  M204V  N236T  M250V  G145R | 0.24 (5/2050) vs. 0.09 (1/1066)  0.15 (3/2050) vs. 0.09 (1/1066)  0.78 (16/2050) vs. 1.40 (15/1066)  2.40 (50/2050) vs. 4.20 (45/1066)  0.15 (3/2050) vs. 0.19 (2/1066)  1.07 (22/2050) vs. 2.35 (25/1066)  1.51 (31/2050) vs. 1.59 (17/1066)  0.83 (17/2050) vs. 0.75 (8/1066)  0.94 (19/2050) vs. 0.29 (3/1066)  2.00 (65/3208) vs. 4.20 (95/2270) | 2.66  1.66  0.55  0.57  0.78  0.45  0.94  1.10  3.24  0.47 |
| 47 Y Eastern European/F on Tenofovir, Genotype D  # 8B | CD56+ | I169T  L180M  A181T/V T184N  A194T  S202  M204V/I N236T  M250 V/I G145R | 0.19 (5/2593) vs. 0.24 (5/2050)  0.03 (1/2593) vs. 0.15 (3/2050)  1.10 (31/2593) vs. 0.78 (16/2050)  2.40 (63/2593) vs. 2.4 (50/2050)  0.19 (5/2593) vs. 0.15 (3/2050)  1.60 (42/2593) vs. 1.00 (22/2050)  1.0 (27/2593) vs. 1.5 (31/2050)  1.00 (27/2593) vs. 0.80 (17/2050)  0.60 (16/2593) vs. 0.90 (19/2050)  ? vs. 2.00 (65/3208) | 0.79  0.20  1.41  1.00  1.26  1.60  0.66  1.25  0.66  - |
